# Re-thinking treatment strategies for febrile neutropenia in paediatric oncology population: the perspective from a developing country

**DOI:** 10.1186/s13027-021-00387-y

**Published:** 2021-06-19

**Authors:** Vinson James, Anand Prakash, Kayur Mehta, Tarangini Durugappa

**Affiliations:** 1 Department of Paediatrics, St. John’s Medical College Hospital, Bengaluru, Karnataka 560034 India; 2 Division of Paediatric Hematology and Oncology, Paediatric Department, St. John’s Medical College Hospital, Bangalore, Karnataka 560034 India; 3 Division of Paediatric Infectious Diseases, Montreal Childrens Hospital, McGill University, Montreal, H3A0G4 Canada

**Keywords:** Febrile neutropenia, Microbiological profile

## Abstract

**Background:**

This study was conducted to evaluate the microbiological profile of bacterial isolates in febrile neutropenia in a pediatric oncology unit, thereby, reviewing the use of restricted antibiotics and need for aggressive medical treatment accordingly.

**Methods:**

A prospective observational study was conducted in a paediatric haemat-oncology division of a tertiary care teaching hospital in southern India from September 2014 to August 2016. One hundred and thirty children with febrile neutropenia were enrolled in the study. Blood cultures were performed using automated system. Cultures from other sites were obtained if needed, based on the clinical profile. Standard antibiotic susceptibility testing was done. Statistical analysis was done using SPSS.

**Results:**

One hundred and thirty children were enrolled for the study. Two hundred and fifty episodes of febrile neutropenia were studied. Three hundred and eighty four cultures were sent and 92 (24%) cultures were positive. There were 48 (52.2%) Gram negative isolates followed by 33 (35.8%) Gram positive isolates, six (6.5%) fungal isolates and five (5.5%) poly-microbial cultures. Lactose fermenting Gram negative bacilli (20 isolates, 31.5%) were the most frequently isolated in the Gram negative group, with *Escherichia coli* being the most common organism (19 isolates, 20.6%). Amongst the Gram positive coagulase negative staphylococcus was the most common (twenty seven isolates, 29%). *Escherichia coli* and Non lactose fermenting gram negative bacteria (NFGNB) had only 36, 25% sensitivity to ceftazidime, respectively. Most Gram negative bacilli were found to have better sensitivity to amikacin (mean: 57%). There was a higher prevalence of extended spectrum beta lactamase producing organisms. Pan drug resistance, Extreme drug resistance and Multi drug resistance was found in three, twenty and thirteen Gram negative isolates respectively.*Escherichia coli* and Klebsiella were often drug resistant. Significantly higher mortality was associated with Gram negative isolates (eight deaths out of the thirteen deaths, 61.5%).

**Conclusions:**

Our results show the importance of surveillance, monitoring resistance frequencies and identifying risk factors specific to each region. Given that significant mortality is attributed to drug resistant Gram negative bacilli, early initiation of appropriate antibiotics to cover for drug resistance is required while formulating empirical antibiotic policies for febrile neutropenia in the oncology units in the developing world.

## Introduction

Keeping abreast of the changes in spectrum of bacterial infections and trends in drug resistance is essential in paediatric oncology units. Intensive chemotherapy in leukemia and other malignancies causes neutropenia and febrile episodes [1]. Neutropenia is defined as an Absolute Neutrophil Count (ANC) < 500/μL or less than 1000/μL with an anticipated decline to less than 500/μL in the next 48-h period. Neutropenic fever is a single oral temperature of 38.3 °C (101 ° F) or a temperature of greater than 38.0 °C (100.4 ° F) sustained for more than 1 h in a patient with neutropenia. Febrile neutropenia is mostly caused by infectious agents [2]. Empirical antibiotic therapy (broad spectrum antibiotic) has been successfully used in the management of febrile neutropenia.

Recent trends in febrile neutropenia in children suggest an emergence of uncommon and drug resistant Gram negative bacilli as the most important cause of morbidity and mortality [3]. Such infections are common in immunocompromised conditions especially malignancies [4]. There is variability in drug resistance patterns among various isolates [5]. So, appropriate empirical antibiotics need to be chosen carefully. Gram positive isolates may have methicillin resistance**.** Many studies show that patients with Gram negative infections have poorer prognosis and higher mortality compared to Gram-positive bacteraemia [6]. Due to higher prevalence of drug resistance and mortality in Gram negative organisms, the treatment regimen chosen for empirical antibiotics is of paramount importance. The changing microbiological spectrum, in febrile neutropenia and the resistance patterns, help guide antibiotic treatment [7].

Audit of febrile neutropenic episodes, would help guide treatment protocols to reduce the mortality and improve outcomes. We conducted a single centre prospective study aimed at identifying the trends in the pattern of microbiological isolates and sensitivity patterns for episodes of febrile neutropenia in the paediatric oncology unit of a tertiary care teaching hospital. The trends that were revealed can help guide optimal antimicrobial therapy.

## Materials and methods

A prospective observational study was conducted at the Division of Paediatric hematology oncology from 1st September 2014 to 31st August 2016. Neutropenia was defined as Absolute Neutrophil Count (ANC) < 500/μL or less than 1000/μL with an anticipated decline to less than 500/μL in the next 48-h period. The criterion for inclusion into the study was children (i.e., less than 18 years) with various malignancies diagnosed with febrile neutropenia. Those with fever, that occurred during the administration of chemotherapy (i.e., less than 24 h) or fever occurring during or within 6 h of transfusion of blood or blood products were excluded. A detailed history regarding fever, type of cancer, chemotherapy regimen, was recorded and physical examination was conducted.

Blood counts and blood cultures were drawn and first line intravenous antibiotics (Ceftazidime and amikacin) were started as per unit protocol, in paediatric emergency room. If a child had a central venous access device, blood cultures were drawn only from that device. Urine culture was done in patients with urinary symptoms, endotracheal tube trap culture was sent of patients intubated during the course of hospitalisation where clinically indicated. Cerebrospinal fluid cultures were examined in the microbiology department in those who developed neurological symptoms during the course of febrile neutropenia. Identification of the pathogens was done by automated technique for blood and manual plate culture technique for urine, cerebrospinal fluid and endotracheal tube trap collection. Standard testing by disc diffusion method was done to find the antibiotic susceptibility. The pathogenic organisms were grown on Mueller-Hinton agar in the presence of various antimicrobial impregnated filter paper disks. The presence or absence of growth around the disks was considered as an indirect measure of the ability of a particular antibiotic to inhibit the organism. The reference for interpretation of sensitive, intermediate resistant and resistant for each antimicrobial was performed by identification of Minimum inhibitory concentration (MIC). Specific MIC values were used as cut off to classify bacteria as susceptible, intermediate or resistant to a specific antibiotic.

Shock was treated as per the surviving sepsis guidelines and such patients were monitored in the Pediatric Intensive Care Unit (PICU) [8, 9]. Antibiotics were stopped once the patient was afebrile for 48 h and if ANC more than 500 /mm^3^ for two consecutive days with no definite site of infection and a negative culture report.

Change to higher antibiotics (second/third line antimicrobials) was considered on non-resolution of fever and**/** or signs of persistent active infections (like rigors or hemodynamic instability) and**/** or based on results of culture/sensitivity reports.

Ceftazidime and amikacin were prescribed as first line antibiotics. In episodes of persisting fever, second line antibiotics (piperacillin**/** tazobactam and amikacin) were started. If there was persistent fever with hemodynamic compromise, a third line drug (meropenem or vancomycin) was initiated till initial blood culture reports were available. Amphotericin B was the anti-fungal of choice. Voriconazole was used for Aspergillus infections. Dengue haemorrhagic fever was suspected if patient presented with signs of tender hepatomegaly, third spacing of fluids and was positive based on dengue serology. Dengue fever was treated based on standard guidelines [10].

The restricted antibiotics for use in this study were colistin, meropenem, vancomycin and tigecycline. Sensitivity patterns were analysed and resistance patterns were mapped. Multi-drug resistance (MDR) was defined as acquired non-susceptibility to at least one agent in three or more antimicrobial categories. Extensive drug resistance (XDR) was defined as non-susceptibility to at least one agent in all but two or fewer antimicrobial categories (i.e., bacterial isolates remain susceptible to only one or two antimicrobial categories). Pandrug resistance (PDR) was defined as non-susceptibility to all agents in all antimicrobial categories [11].

The study was approved by The St. John’s Medical College Institutional Ethics Committee in September 2014 and consent of guardians was taken before including the children into the study.

### Statistical analysis

Descriptive analysis was done by calculating frequencies, mean values and percentages. Statistical analysis was done by using SPSS 22.0 for windows version. Chi-square test was used for the comparison of categorical variables. *P*-value less than 0.05 were considered statistically significant.

## Results

**Fig. 1:**
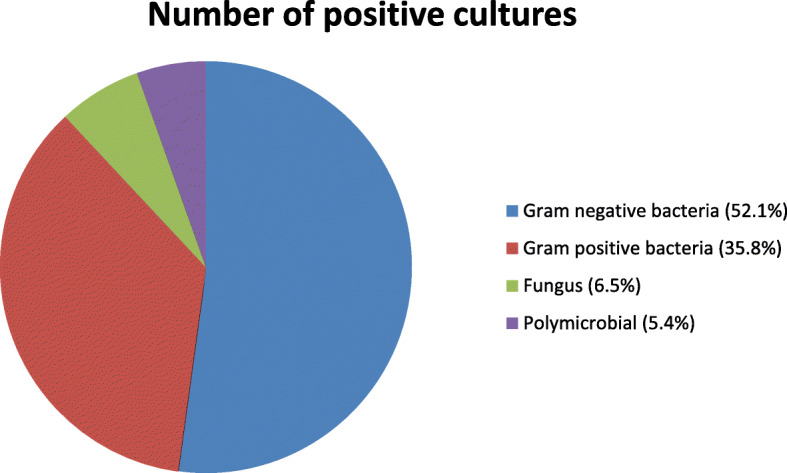
Gram staining pattern

A total of 130 children (250 episodes of febrile neutropenia) were included in this study. Male to female ratio was 1.7: 1. Mean age at presentation was 6.5 years (range: 6 months − 15 years, standard deviation: 4.29 years). Mean duration of fever was 72 h (range: 2–20 days, standard deviation: 3.09 days). Acute Lymphoblastic Leukaemia (ALL) was the most common malignancy noted at 186 episodes (74%), followed by Acute Myeloid Leukaemia (AML) at 47 episodes (19%) followed by Lymphoma, Ewing’s sarcoma and rhabdomyosarcoma. Respiratory symptoms (13%) were the most common presentation, followed by musculoskeletal symptoms (11%). Nine percent of the episodes had features of shock and were admitted to paediatric intensive care unit. No obvious focus of infection was noted in 57% episodes. During the course of hospitalization, all the episodes included in the study had ANC < 500 and ANC < 200 was observed in 50% of the episodes. The average number of cultures examined per episode of neutropenia was 1.536. A total of 300 and 84 cultures were examined, of which 92 (24%) were positive. Two hundred and forty six blood cultures (64%) were examined, of which 65 (17%) were positive. Once the blood cultures were examined, empiric antibiotics were started. In case of no clinical improvement or**/** and worsening, further culture samples were taken under strict asepsis. A total of 92 (24%) urine cultures, 26 (6.7%) endotracheal trap aspirate cultures and twenty (5%) cerebrospinal fluid cultures were obtained. Most common organisms isolated were Gram Negative Bacilli (GNB) (forty eight isolates, 52.2%), followed by Gram positive cocci (thirty three isolates, 35.8%), fungi (six isolates, 6.5%) and 5 poly-microbial cultures (5.5%) in 92 culture positive episodes. (Fig. 1) Amongst the GNB, the lactose fermenters (twenty nine isolates, 31.5%) predominated with *Escherichia coli* being the most common (nineteen isolates, 20.6%) followed by Klebsiella (ten isolates, 10.8%). NFGNB constituted nineteen isolates, (20.6%) including Pseudomonas (six isolates, 6.5%). Amongst Gram positive, Coagulase negative staphylococcus (CONS) was the most common (29%), followed by Streptococcus and Enterococcus.

The sensitivity patterns have been displayed in tables. (Tables 1 and 2) We noticed that almost all Gram positive isolates in our cultures showed excellent response to first and second line antibiotics with CONS showing sensitivity of 96% to amikacin, 100% to ceftazidime, piperacillin-tazobactam, meropenem, vancomycin and colistin. Streptococci and Enterococci had less susceptibility to first line antibiotics but responded well to vancomycin and meropenem. The Gram negative bacilli on the other hand had poor response to ceftazidime and relatively better response to amikacin. Lactose fermenters like *Escherichia coli* had sensitivity to ceftazidime in only 36% and NFGNB was sensitive in 25%. *Escherichia coli* strain was sensitive to amikacin in 71% episodes and Pseudomonas had excellent susceptibility (100%) as well. Klebsiella and NFGNB (other than Pseudomonas) had relatively poor sensitivity to amikacin. All Gram negative bacilli both lactose-fermenters and non-fermenters showed a mean of sensitivity of only 16.7, 24.7 and 33.7 to piperacillin-tazobactom, ceftazidime and meropenem**,** respectively. NFGNB (other than Pseudomonas) and Klebsiella were 44% sensitive to amikacin. Pseudomonas and *Escherichia coli* had a sensitivity of 100 and 71%, respectively to amikacin. The mean of sensitivity of all Gram negative bacilli to amikacin was 57%. NFGNB (other than Pseudomonas) had a sensitivity of 68% to colistin. *Escherichia coli* and Klebsiella showed even higher sensitivity of 90 and 80%, respectively to colistin.

**Table 1:** Sensitivity pattern of Gram positive organisms

Organism	Number (% out of total culture positive)	Ceftazi-dime Sensi-tivity	Amika-cin Sensi-tivity	Piperacillin Tazobactam Sensi--tivit	Meropenem Sensi-tivity	Colistin Sensi-tivity	Tigecycline Sensi-tivity	Vancomycin Sensi-tivity
CONS	27 (29)	100%	96%	100%	100%	100%	83%	100%
*Staphylococcus aureus*	1 (1)	0	100%	100%	100%	100%	100%	100%
Streptococcus	2 (2)	50%	33%	50%	100%	50%	50%	100%
Enterococcus	3 (3.2)	0	0	0	0	0	0	100%

*CONS* Coagulase negative staphylococcus aureus

**Table 2:** Sensitivity pattern of Gram negative organisms

Organism	Number (% out of total culture positive)	Ceftazidime Sensitivity (%)	Amikacin Sensi-tivity	Piperacillin-Tazobactam Sensitivity	Meropenem Sensi-tivity	Colistin Sensi-tivity	Tigecycline Sensi-tivity
NFGNB (except pseudomonas)	13 (14)	25	44%	20%	29%	68%	43%
Pseudomonas	6 (6.5)	25	100%	0	20%	0	0
*Escherichia coli*	19 (20.6)	36	71%	21%	43%	90%	50%
Klebsiella	10 (11)	13	44%	22%	44%	80%	0
**Mean of sensitivity**		24.7	57%	16.7	33.7	65	29

*NFGNB* Non lactose fermenting gram negative bacteria

**Fig. 2:**
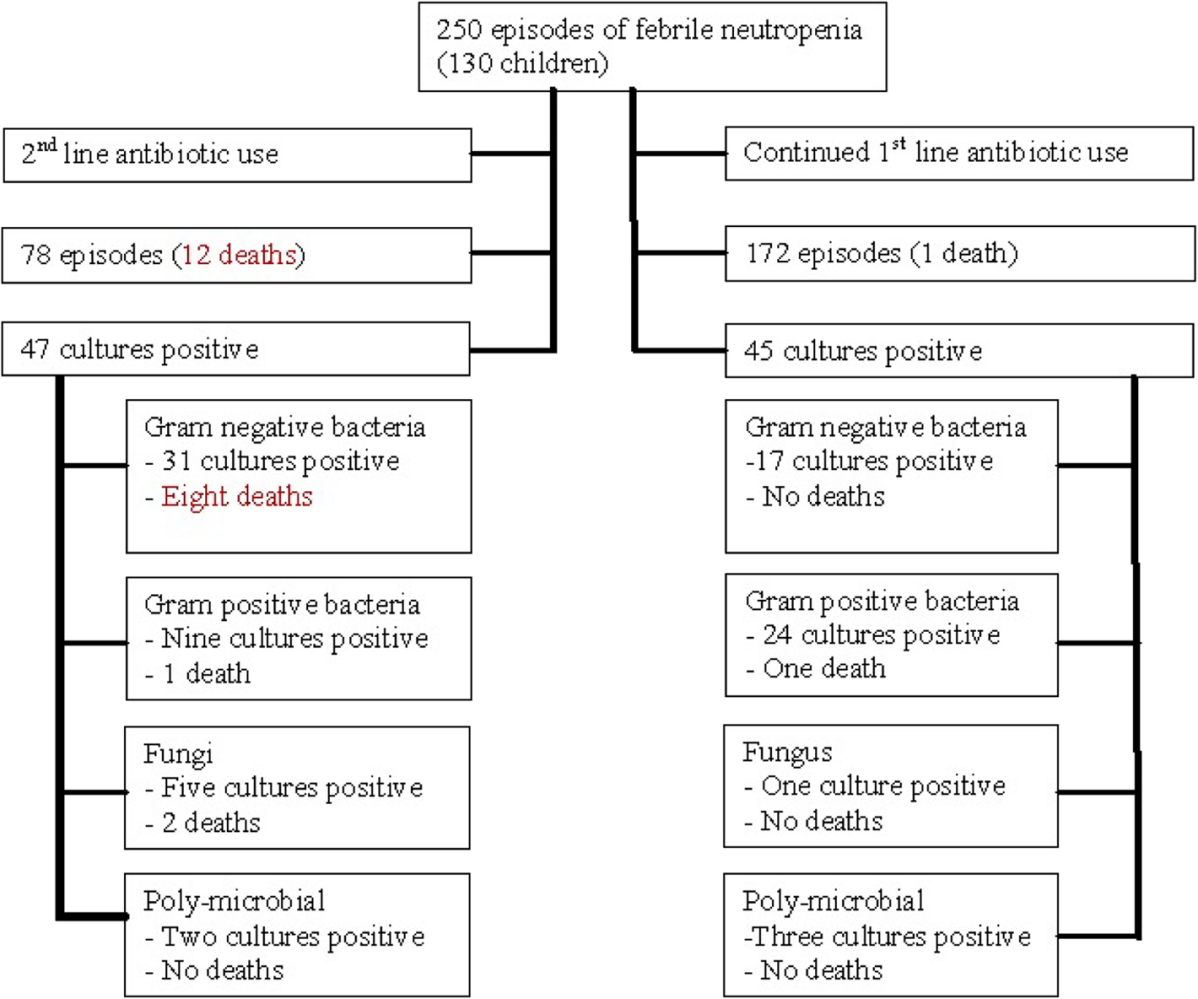
Breakdown of two hundred and fifty episodes based on culture sensitivity

First line antibiotics (ceftazidime and amikacin) were started for all the episodes, however, 78 (31%) episodes required second line antibiotics. These 78 episodes included 47 positive cultures (51% of all positive cultures) and 12 deaths (92% of all deaths). Of the 47 positive cultures within this group 31 (66%) belonged to Gram negative group, while only nine (19%) belonged to Gram positive group. Episodes with Gram negative isolates were more likely to require second line antibiotics (51%). (Table 3) Out of the 12 deaths in this group, eight (61% of all deaths) died due to Gram negative bacteraemia. (Fig. 2).

**Table 3:** Need for second line antibiotics for Gram negative Bacilli versus others

	Use of second line antibiotics (% of all culture positive episodes)	Use of first line antibiotics (% of all culture positive episodes)
Gram negative bacilli	31 (34)	17 (18.5%)
Others	16 (17)	28 (30%)

*significant at the level of *p* < 0.05

**Fig. 3:**
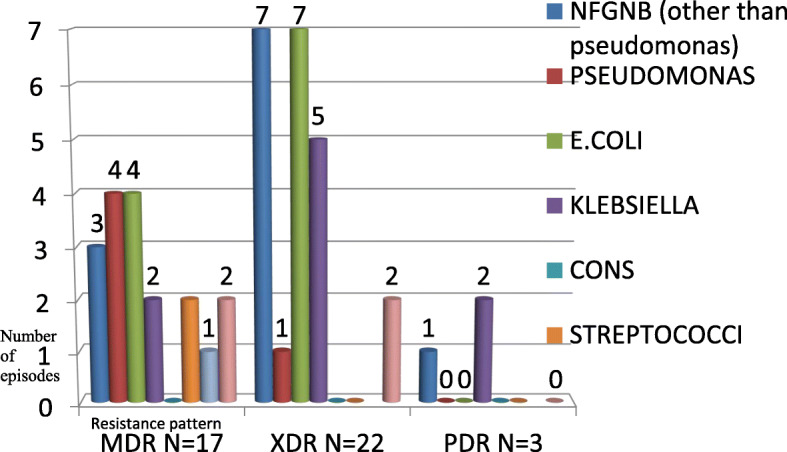
Common drug resistant organisms

Pan drug resistance was found in three Gram negative cultures, associated with two deaths. Extreme drug resistance was seen in twenty Gram negative isolates, associated with five deaths. Multi drug resistance was seen in thirteen Gram negative isolates, associated with one death. Most drug resistance (i.e., MDR, XDR and PDR, a total of 36) was noticed in the Gram negative group. (Table 4) *Escherichia coli* and Klebsiella were found to have more resistant isolates and highest mortality. (Fig. 3).

**Table 4:** Drug Resistance (MDR, XDR and PDR) in Gram negative bacilli versus others

	Sensitive (% of all culture positive episodes)	Resistance (% of all culture positive episodes)
Gram negative bacilli	12 (13)	36 (39)
Gram positive cocci	27 (29)	06 (6.5)

*significant at the level of *p* < 0.05

Much higher mortality was seen in episodes with Gram negative isolates and especially higher in drug resistant isolates. (Table 5) Amongst those requiring second line antibiotics, 12 deaths were reported of which eight deaths were due to Gram negatives, one death was due to Gram positive and two deaths due to fungal infection. In the group that required only first line antibiotic, most common isolate was Gram positive cocci (twenty four isolates) while Gram negative bacilli relatively lesser (seventeen isolates). Only one death was reported in this group. There was only one death due to viral cause which was due to dengue haemorrhagic fever.Irfan et

**Table 5:** Mortality in Gram negative bacilli versus others

	Deaths (percentage of deaths/ %of culture positive isolates)	Alive (%of culture positive isolates)
Gram negative bacilli	8 (61 / 8.7)	23 (25)
Others	4 (30.5/ 4.3)	41 (44.5)

*significant at the level of *p* < 0.05

## Discussion

The data from western countries show a prevalence of Gram positive organisms in those countries [12, 13]. However, in developing countries**,** like India, there has been a rising trend of Gram negative bacteremia [14]. In our study, GNB accounted for 52% of the episodes. Our study reinforces this recent trend and demonstrates that the switch to Gram positive cocci amongst the western countries may be just limited to the developed countries [15]. Cattaneo et al., also stated an evidence of epidemiological shift from Gram positive to GNB in febrile neutropenic patients with *Escherichia coli* being the most frequent organism [16]. There have been other reports of emergence of Gram negative bacilli, predominantly Pseudomonas and Enterobacteriaceae [17].

Since febrile neutropenia is a clinical emergency which needs to be addressed timely, empiric antibiotics are administered as soon as possible, even before culture sensitivity results are available, which may take 2–3 days. A delay of more than 48 h in the administration of appropriate antibiotics may result in a mortality rate as high as 50% [18]. In previous studies**,** mortality has been variably reported from 5 to 39% in other developing countries [19, 20]. Thirteen patients died during the study period (10% of the total patients with febrile neutropenia). The interesting fact we noted was that 61.5% of the deaths were due to Gram negative bacteremias.

In our study, initial empirical antimicrobial treatment was found to be appropriate in only 35% cases, probably due to lower susceptibility to ceftazidime. Due to higher load of drug resistant (MDR, PDR, XDR) bacilli, febrile neutropenic patients with Gram negative bacteremia usually present with rapid disease onset and multiple complications. Similar presentations were also noticed by Babu et al., in a study conducted in southern India [21]. Clinically worsening patients required second line antibiotics. This group, also had, significantly more deaths (61% of all deaths) as well as more pediatric intensive care unit (PICU) admissions. All patients who died had required PICU admission. Irfan et al. [22] had described emergence of carbepenem resistant extended spectrum beta lactamases. Similar to this study**,** we also had a predominance of piperacillin-tazobactam and meropenem resistant organisms. Emergence of carbepenem resistant extended spectrum beta lactamase organisms have also been reported by Micozzi et al. [23] Few recent studies in China have also reported increasing requirement of higher antibiotics (almost 70%) in neutropenic patients, similar to our study, wherein a total of 78 episodes (47 out of 92 culture positives) required escalation i.e., in nearly 51% cases. A significant proportion in this group was GNB, which constituted 31 (66%) of these cases.

Three out of the total GNB that required second line antimicrobials were Pan drug resistant (PDR) (resistant to all drugs). While 20 were classified to be extensively drug resistant. Eight out of the total 13 deaths were caused by these highly resistant bacilli. Hence, the importance of knowing the locally prevalent pathogens and their susceptibility pattern cannot be emphasized enough.

We found a higher prevalence of extended spectrum beta lactamase producing organisms with poor sensitivity to first and second line antibiotics in a substantial number of patients in our hospital set up. Although relatively good sensitivity was noted with amikacin, monotherapy with amikacin alone will not suffice and it is advisable to add higher antibiotics like colistin earlier, in case of clinical worsening.

Epidemiology and resistance patterns in local hospitals should be considered when choosing empirical antibiotic treatments as the optimal treatment strategy might vary in different local settings.

Since most of the oncology units, even in developing countries have Bactec automated culture systems, which can detect the involved organisms sooner, we propose that early escalation to higher antibiotics be promoted, based on local culture sensitivity pattern, especially in patients with Gram negative bacteremia and clinical deterioration.

## Conclusions

In view of higher prevalence of Gram negative isolates and emergence of multi-drug resistance**,** frequent audits of resistance patterns should guide choice of antimicrobials in febrile neutropenia management. Aminoglycosides along with broad spectrum antibiotics based on local culture sensitivity profiles should be included for upfront empiric antibiotic coverage for children with febrile neutropenia especially in the context of drug resistance in the developing world.

Early administration of restricted antibiotics may need to be considered early if automated culture systems reveal Gram negative bacteremia especially if patients are unstable. (earliest detection of the organism) to prevent complications like sepsis and multi-organ failure.

## Data Availability

All data generated or analysed during the study are included in this published article and in supplementary files.

## Abbreviations

ALL: Acute Lymphoblastic Leukemia
AML: Acute Myeloid Leukemia
ANC: Absolute Neutrophil Count
CONS: Coagulase Negative Staphylococcus
GNB: Gram Negative Bacilli
GPC: Gram Positive Cocci
ICU: Intensive Care Unit
MDR: Multi Drug Resistant
NFGNB: Non- lactose Fermenting Gram Negative Bacilli
PDR: Pan Drug Resistant
PICU: Pediatric Intensive Care Unit
UTI: Urinary Tract Infection
XDR: Extensively Drug Resistant.
